# Page kidney: A rare cause of secondary hypertension

**DOI:** 10.4102/sajr.v23i1.1762

**Published:** 2019-09-23

**Authors:** Ilonka Warnich, Mark Nicolaou, Zelia Sofianos, Jacobus A. Pienaar, Jacob Varghese

**Affiliations:** 1 Department of Diagnostic Radiology, University of the Witwatersrand, Johannesburg, South Africa; 2 Department of Diagnostic Radiology, Klerksdorp/Tshepong Hospital Complex, Klerksdorp, South Africa

**Keywords:** Page kidney, young, hypertension, secondary hypertension, subcapsular collection

## Abstract

Page kidney is a rare phenomenon that can present with hypertension. The presence of a subcapsular perirenal collection causes parenchymal compression leading to renal hypoperfusion. Subsequent activation of the renin–angiotensin–aldosterone system results in an increase in systemic blood pressure. The causes of renal subcapsular collections are varied, with most cases being secondary to post-traumatic haematomas. We present the case of a young hypertensive patient, treated as primary hypertension with persistently uncontrolled blood pressures. This was despite good treatment adherence. On further investigation, imaging identified the presence of bilateral subcapsular collections. This case illustrates the importance of a thorough workup in a young hypertensive patient with refractory hypertension. Given that Page kidney is curable, timeous intervention can save the patient from unnecessary medications and the morbidity of uncontrolled blood pressures.

## Introduction

Page kidney is an entity that few doctors have come across and is rarely considered as part of the differential diagnosis in a young hypertensive patient. However, it is a crucial diagnosis to make, as it is a potentially curable cause of secondary hypertension.

## Case presentation

A 23-year-old female presented to the emergency department (ED) with an acute history of neck stiffness and headache, not responding to analgesia. She had been diagnosed with hypertension at a local clinic and was referred to the ED for further workup and management. On initial clinical examination, blood pressure was noted to be 187/117 mmHg. The patient was found to have photophobia, neck stiffness and disorientation. She was worked up for possible meningitis or a subarachnoid haemorrhage. Both conditions were subsequently excluded after cerebrospinal fluid (CSF) biochemistry and computed tomography (CT) brain imaging, respectively. The patient’s renal function was noted to be normal at this stage. During her initial admission, her blood pressure remained elevated despite appropriate therapy and she was later discharged on anti-hypertensive treatment.

The patient then re-presented 4 months later and was admitted as a hypertensive urgency with a blood pressure of 178/118 mmHg. On physical examination, bilateral flank masses were palpated. Further systematic examination revealed no other abnormalities. The patient’s blood results showed deterioration in renal function. The patient’s blood pressure was eventually controlled with a combination of three anti-hypertensive agents.

**FIGURE 1:**
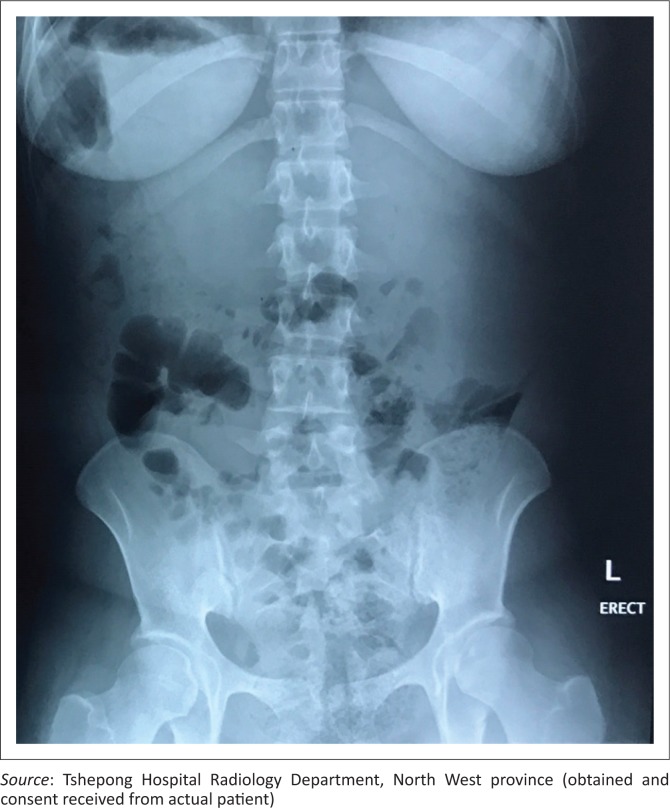
Erect abdominal radiograph demonstrating fullness of bilateral renal fossae with obscuration of the psoas outline suggestive of renal masses. Associated mass effect evidenced by inferior displacement of adjacent bowel loops.

**FIGURE 2:**
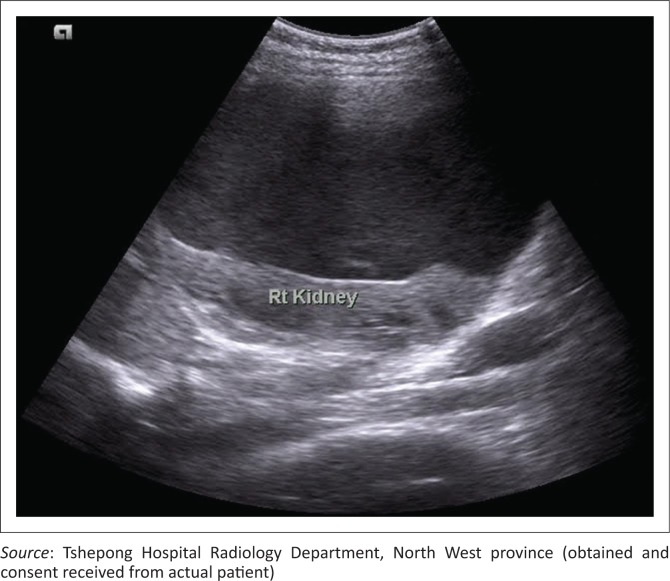
Sagittal ultrasound image of the right kidney depicting a large perirenal hypoechoic fluid collection with hyperechoic internal echoes compatible with complex fluid. Associated medial displacement and compression of the right kidney. Similar findings were seen on the contralateral side (not shown)

**FIGURE 3:**
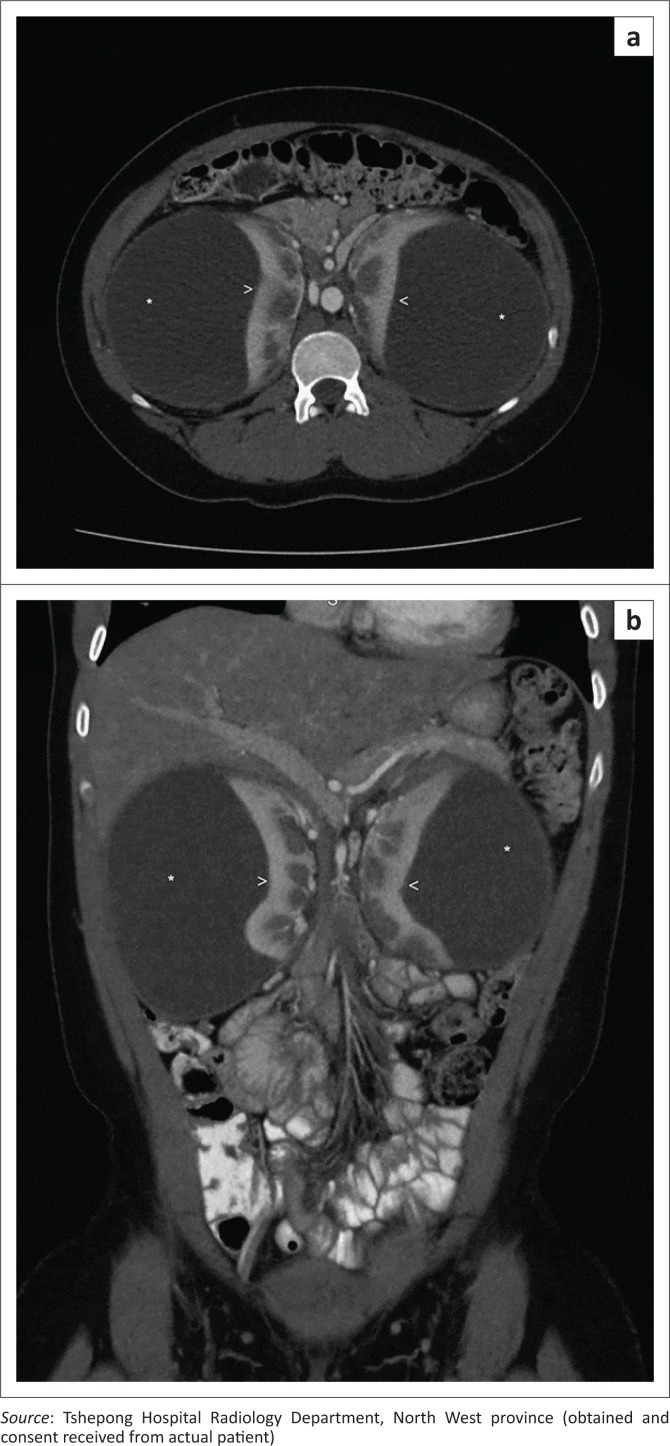
(a, b) Contrast-enhanced axial and coronal computed tomography of the abdomen demonstrating large bilateral subcapsular collections (stars) causing compression of the renal parenchyma (arrowheads).

An erect abdominal radiograph demonstrated fullness in bilateral renal fossae with obscuration of the psoas outlines, as shown in Figure 1. A bedside ultrasound performed by the attending clinicians revealed bilateral encapsulated renal cysts. A working diagnosis of polycystic kidney disease was made. A subsequent ultrasound was then repeated in the radiology department, showing bilateral subcapsular renal collections with low-level internal echoes (Figure 2). A contrast CT study of the abdomen was performed and demonstrated large bilateral subcapsular perirenal collections with the fluid measuring 24 Hounsfield units (Figure 3a and 3b). Additional findings included compression and anatomical distortion of the kidneys.

The aforementioned imaging features were in keeping with bilateral perirenal haematomas. A differential diagnosis of bilateral renal lymphangiomatosis was entertained. However, given the complex nature of the fluid on ultrasound and elevated Hounsfield units on CT, this diagnosis was less likely. On further clinical evaluation, the patient denied any history of prior trauma. An underlying coagulopathy was excluded. The patient was subsequently referred to a tertiary institution for further management.

### Ethical consideration

Informed consent from the patient was obtained; permission from the CEO of the hospital was also obtained.

## Discussion

The workup of a young hypertensive patient, defined as hypertension below the age of 30, has traditionally consisted of extensive investigations to exclude a secondary cause. However, the prevalence of hypertension among the young has rapidly increased, doubling over the past decade, and in most cases a diagnosis of primary hypertension is made.^1^ Because of the high demand on healthcare costs, it has been recommended that the routine workup of these patients is limited to baseline investigations. Special tests, such as renal imaging, should be directed and reserved for patients with a clinical suspicion of an underlying cause.^1^ In most institutions, renal ultrasound is still considered a baseline investigation; however, it is not always readily available. Therefore, as in our case, young patients are frequently managed as primary hypertension with a resultant delay in the diagnosis of a secondary cause.

Irvine Page first described the phenomenon of Page kidney in 1939 after wrapping canine kidneys in cellophane and observing a consequent rise in blood pressure.^2^ Engle and Page documented the first clinical case of Page kidney in 1955, when a young American football player was diagnosed with a subcapsular renal haematoma and concomitant hypertension. Following a nephrectomy, the patient’s blood pressure normalised.^3^

The presumed pathogenesis of hypertension is by sustained renal parenchymal compression with altered small vessel haemodynamics. The resultant hypoperfusion and microangiopathic ischaemia then trigger the renin–angiotensin–aldosterone system (RAAS), leading to salt and water retention with a rise in systemic blood pressure.^3^ This mechanism of hyper-reninaemic hypertension has been likened to Goldblatt’s model of renovascular hypertension, which is caused by compression or stenosis of the major renal vasculature.^4^

Many causes for Page kidney exist. The external renal compression is most often in the form of a subcapsular collection. The subcapsular space is a potential perinephric space where fluids, such as blood, pus, urine, lymph, exudates or transudates, can collect.^5^ These occur secondary to various underlying conditions, which will guide the workup of the patient.

The traumatic or iatrogenic subcapsular haematoma has been shown to be the most common cause of Page kidney.^6^ Previously, most cases were attributed to football or non-sports-related blunt abdominal trauma, referred to as a classical Page kidney.^7^ Analysis of cases reported after 1991 revealed an etiological shift towards the iatrogenic subcapsular haematoma.^8^ These most often occurred as complications of renal allograft biopsies but also after extracorporeal shock wave lithotripsy or ureteronephroscopic procedures.^3^

Non-traumatic causes of page kidney are uncommon with isolated cases reported in the literature. These include spontaneous renal haemorrhage secondary to underlying tumours, arteriovenous malformations, cyst rupture, glomerulonephritis or vasculitis. Subcapsular urinomas and lymphatic collections have also been described. In some cases, Page kidney may be idiopathic.^6,7^

It is important to keep in mind that there may be a significant time lapse between the traumatic insult and the diagnosis of hypertension, with intervals from days to decades having been reported. The traumatic event could even occur unnoticed. The presentation can range from non-specific signs and symptoms with an insidious onset, to an acute episode of hypertensive urgency or emergency.^4^ Page kidney should always be a consideration in the young hypertensive patient presenting with a renal mass or previous abdominal trauma.^9^ Renal ultrasound and contrast-enhanced CT are generally sufficient in the diagnosis of Page kidney.^6^

In cases of spontaneous renal haemorrhage, an important underlying cause to consider as part of the workup will include neoplastic lesions. Of these, angiomyolipomas and renal cell carcinomas are the most common.^10^ A renal mass may not always be evident on the initial imaging and in some cases is only diagnosed on follow-up imaging after resolution of the haematoma.^5^ If no tumour is evident on CT, selective renal angiography can be valuable to assess for vascular diseases, such as polyarteritis nodosa, arteriovenous malformations or renal artery aneurysms.^5^ Therapeutic embolisation can then be considered as part of the management. Angiographic evaluation should especially be considered in cases of recurrent non-traumatic Page kidney.^4^ Spontaneous renal haemorrhage can also be caused by coagulation disorders, which should form part of the diagnostic workup.^10^

Bilateral renal lymphangiomatosis, also referred to as lymphangiectasia, is an uncommon cause of Page kidney. The typical imaging findings are bilateral subcapsular low-density fluid collections, with Hounsfield units ranging from 0 to 10. Renal ultrasound findings can include multiseptated perirenal fluid collections with or without peripelvic cysts.^11^ As an ancillary investigation, lymphoscintigraphy will reveal perirenal lymphatic leakage. Aspiration and biochemistry of the perirenal fluid can assist in the diagnostic workup.^11^

Previously, the management of classical Page kidney involved radical nephrectomy or open surgery. With medical advances and improvements in anti-hypertensive drugs, especially those directed against the RAAS, management has shifted to a more conservative approach.^8^ There is still no standardisation in the definitive treatment of Page kidney, which will depend on various patient factors, as well as the underlying cause.^3^

Conservative management of subcapsular haematomas, with follow-up imaging to ensure resolution, can be successful in some cases.^3^ Surgical intervention, however, is often necessary. The two main goals are to decompress the kidney by evacuating the haematoma and to remove the fibrocollagenous pseudocapsule, which can form in chronic cases.^12^ Recent case reports have shown minimally invasive procedures such as laparoscopic- or radiologic-assisted percutaneous drainage to be viable therapeutic alternatives.^3^ Percutaneous drainage is thought to have higher success rates in subcapsular haematomas of less than 3 weeks duration. More chronic organised haematomas often require invasive procedures for adequate evacuation.^3^ In cases of lymphangiomatosis, the preferred management includes percutaneous drainage with injection of sclerosing agents.^11^

In some cases of Page kidney, patients may require chronic anti-hypertensive treatment, regardless of the underlying cause and resolution thereof. This is partly because of the fact that there is often a delay in diagnosis with subsequent perinephric scarring.^7,12^

## Conclusion

This case illustrates the need for a comprehensive workup of the young hypertensive patient and the importance of Page kidney as a secondary, potentially reversible cause.

## References

[1] Mangena P, Chb MB, Sa FCP, Saban S, Chb MB, Sa F An approach to the young hypertensive patient. SAMJ. 2016;106(1):36–38. 10.7196/SAMJ.2016.v106i1.10329 26933708

[2] Page IH The production of persistent arterial hypertension by cellophane perinephritis. JAMA. 1939;113(23):2046–2048. 10.1001/jama.1939.02800480032008

[3] Kobel MC, Nielsen TK, Graumann O Acute renal failure and arterial hypertension due to subcapsular haematoma: Is percutaneous drainage a feasible treatment? BMJ Case Rep [serial online]. 2016[cited 2019 May 18]. Available from: 10.1136/bcr-2015-212769 PMC4735349 26783007

[4] Kenis I, Werner M, Nacasch N, Mbbs K Recurrent non-traumatic page kidney. IMAJ. 2012;14(7):452–453. 22953625

[5] Haddad MC, Hawary MM, Khoury NJ, Abi-fakher FS, Ammouri NF, Al-kutoubi AO Radiology of perinephric fluid collections. Clin Radiol. 2002;57(5):339–346. 10.1053/crad.2001.0854 12014928

[6] Arslan S ScienceDirect bilateral nontraumatic recurrent Page kidney Radiol Case Rep [serial online]. Elsevier Inc; 2017[cited 2019 May 18];12(3):511–513. Available from: 10.1016/j.radcr.2017.05.003 PMC5552010 28828114

[7] Smyth A, Collins CS, Thorsteinsdottir B Page Kidney: Etiology, renal function outcomes and risk for future hypertension. J Clin Hypertens. 2012;14(4):216–221. 10.1111/j.1751-7176.2012.00601.x PMC8108801 22458742

[8] Dopson SJ, Jayakumar S, Velez JCQ Page kidney as a rare cause of hypertension: Case report and review YAJKD [serial online]. National Kidney Foundation, Inc.; 2009[cited 2019 May 18];54(2):334–339. Available from: 10.1053/j.ajkd.2008.11.014 19167799

[9] Sokhal AK, Prakash G, Saini DK, Singh K Page kidney: A rare but surgically treatable cause of hypertension. Saudi J Kidney Dis Transpl. 2018;29(1):193–197. 10.4103/1319-2442.225183 29456229

[10] Ahn T, Roberts MJ, Navaratnam A, Hirst J, Wood S Recurrent spontaneous renal haemorrhage due to polyarteritis nodosa: A medical cause for a surgical problem. ANZ J Surg[serial online]. 2017[cited 2019 May 18]. 10.1111/ans.13914 28239941

[11] Choudhury S, Sridhar K, Pal D Renal lymphangiectasia treated with percutaneous drainage and sclerotherapy. Int J Adolesc Med Health[serial online]. 2017[cited 2019 May 18]. Available from: 10.1515/ijamh-2017-0024 28598807

[12] Davies MC, Perry MJ, Georges S Urological management of ‘Page kidney’. BJU Int. 2006;98(5):943–944. 10.1111/j.1464-410X.2006.06432.x 17034594

